# Hairy gene homolog increases nasopharyngeal carcinoma cell stemness by upregulating *Bmi-1*

**DOI:** 10.18632/aging.204742

**Published:** 2023-05-22

**Authors:** Ye Lei, Hong-Fen Shen, Qi-Wen Li, Sheng Yang, Hong-Ting Xie, Xu-Feng Li, Mei-Ling Chen, Jia-Wei Xia, Sheng-Chun Wang, Guan-Qi Dai, Ying Zhou, Ying-Chun Li, Shi-Hao Huang, Dan-Hua He, Zhi-Hao Zhou, Jin-Ge Cong, Xiao-Lin Lin, Tao-Yan Lin, Ai-Bing Wu, Dong Xiao, Sheng-Jun Xiao, Xin-Ke Zhang, Jun-Shuang Jia

**Affiliations:** 1 School of Basic Medical Sciences, Southern Medical University, Guangzhou 510515, China; 2 Laboratory Animal Center, Southern Medical University, Guangzhou 510515, China; 3 Guangzhou Southern Medical Laboratory Animal Sci and Tech Co. Ltd., Guangzhou 510515, China; 4 School of Laboratory Medicine and Biotechnology, Southern Medical University, Guangzhou 510515, China; 5 School of Basic Medical Sciences, Guangxi Medical University, Nanning 530000, China; 6 The Third People’s Hospital of Kunming (The Sixth Affiliated Hospital of Dali University), Kunming 650041, China; 7 Department of Pathology, School of Basic Medicine, Guangdong Medical University, Dongguan 523808, China; 8 Cancer Center, Integrated Hospital of Traditional Chinese Medicine, Southern Medical University, Guangzhou 510315, China; 9 Department of Pharmacy, Nanfang Hospital, Southern Medical University, Guangzhou 510515, China; 10 Central People’s Hospital of Zhanjiang, Zhanjiang 524000, China; 11 National Demonstration Center for Experimental Education of Basic Medical Sciences, Southern Medical University, Guangzhou 510515, China; 12 Department of Pathology, The Second Affiliated Hospital of Guilin Medical University, Guilin 541199, China; 13 Department of Pathology, State Key Laboratory of Oncology in South China, Collaborative Innovation Center for Cancer Medicine, Sun Yat-sen University Cancer Center, Guangzhou 510060, China

**Keywords:** nasopharyngeal carcinoma (NPC), Bmi-1, HRY, cell proliferation, migration

## Abstract

B-cell-specific Moloney murine leukemia virus integration site 1 (Bmi-1) is overexpressed in various cancer types. We found that *Bmi-1* mRNA levels were elevated in nasopharyngeal carcinoma (NPC) cell lines. In immunohistochemical analyses, high Bmi-1 levels were observed in not only 5 of 38 non-cancerous nasopharyngeal squamous epithelial biopsies, but also in 66 of 98 NPC specimens (67.3%). High Bmi-1 levels were detected more frequently in T3-T4, N2-N3 and stage III-IV NPC biopsies than in T1-T2, N0-N1 and stage I-II NPC samples, indicating that Bmi-1 is upregulated in advanced NPC. In 5-8F and SUNE1 NPC cells, stable depletion of Bmi-1 using lentiviral RNA interference greatly suppressed cell proliferation, induced G1-phase cell cycle arrest, reduced cell stemness and suppressed cell migration and invasion. Likewise, knocking down Bmi-1 inhibited NPC cell growth in nude mice. Both chromatin immunoprecipitation and Western blotting assays demonstrated that Hairy gene homolog (HRY) upregulated Bmi-1 by binding to its promoter, thereby increasing the stemness of NPC cells. Immunohistochemistry and quantitative real-time PCR analyses revealed that HRY expression correlated positively with Bmi-1 expression in a cohort of NPC biopsies. These findings suggested that HRY promotes NPC cell stemness by upregulating Bmi-1, and that silencing Bmi-1 can suppress NPC progression.

## INTRODUCTION

Nasopharyngeal carcinoma (NPC) is a type of squamous cell carcinoma that originates in the mucosal epithelium of the nasopharynx [1–3]. NPC is prevalent in south China, especially Guangdong province, occurring in roughly 20 per 100,000 people annually [1–3]. Due to the deep location and vague symptoms of NPC, most patients exhibit relatively advanced disease during their initial diagnosis, including local invasion and early distant metastases, so the prognosis of NPC tends to be poor [1–3]. Thus, it is essential to determine the molecular pathways involved in the pathogenesis of NPC so that patients can be diagnosed early, receive an accurate prediction of their prognosis and be treated with novel therapeutic strategies.

B-cell-specific Moloney murine leukemia virus integration site 1 (Bmi-1), a widely expressed nuclear protein and proto-oncogene, is a catalytic subunit of Polycomb repressive complex 1 [4–6]. Bmi-1 is required to maintain and promote the self-renewal of mouse adult stem cells such as hematopoietic, small intestinal, lung, prostate, neural, breast and dental pulp stem cells [4–8]. Bmi-1 suppresses the differentiation of adult stem cells and precursor cells, whereas knocking out Bmi-1 has been shown to induce the differentiation of these cells [4–8]. In addition, Bmi-1 promotes cell senescence, immortalization, transcription initiation and chromatin agglutination-related protein interactions [4–6].

Moderate Bmi-1 expression is necessary for development, whereas abnormally high Bmi-1 expression has been linked with the oncogenesis, development and prognosis of various tumor types, including glioma, colorectal cancer, breast cancer and prostate cancer [4–6]. Bmi-1 is also needed for the maintenance and self-renewal of cancer stem-like cells/tumor-initiating cells in leukemia [9–11], colorectal cancer [12], liver cancer [13], glioma [14, 15], breast cancer [16, 17], prostate cancer [18, 19], head and neck squamous cell carcinoma [20], and medulloblastoma [21]. Bmi-1 overexpression was shown to immortalize nasopharyngeal epithelial cells [22] and trigger their epithelial-mesenchymal transition (EMT) [23]. On the other hand, Bmi-1 silencing was reported to promote apoptosis in NPC cells, thereby sensitizing them to chemotherapeutic and radiotherapeutic treatments [24–27]. Moreover, Bmi-1 antibody as a potential marker of NPC may be rational, and could have diagnostic and prognostic value [28].

Hairy gene homolog (HRY) is the mammalian homolog of the Drosophila hairy gene [29], and is a member of the hairy and enhancer of split (HES1-7) gene family [30]. HRY is often used as a stem cell marker, and is necessary for the self-renewal of hematopoietic stem cells [31], small intestinal stem cells [32, 33], melanin stem cells [34] and pancreatic stem cells [35]. HRY is highly expressed in various tumor tissues [36, 37], and is required to maintain the stemness of cancer stem cells [38, 39]. Overexpression of miR-199b was found to downregulate *HRY*, thereby reducing the number of cancer stem cells in medulloblastoma [40]. On the other hand, tumor necrosis factor alpha was shown to increase the content of cancer stem cells in oral squamous cell carcinoma by activating Notch-Hes1 [41]. Moreover, HRY was reported to promote tumor development/progression and maintain cancer stem cell stemness in colon cancer [42]. The Notch signaling pathway was found to enhance the therapeutic resistance of cancer stem cells in lung cancer and ovarian cancer [43, 44].

In the present study, we investigated the influence of Bmi-1 on the proliferation, stemness, motility and invasion of NPC cells, and explored the molecular pathways underlying these effects.

## RESULTS

### High Bmi-1 expression is common among clinical NPC tissue samples

**Figure 1:**
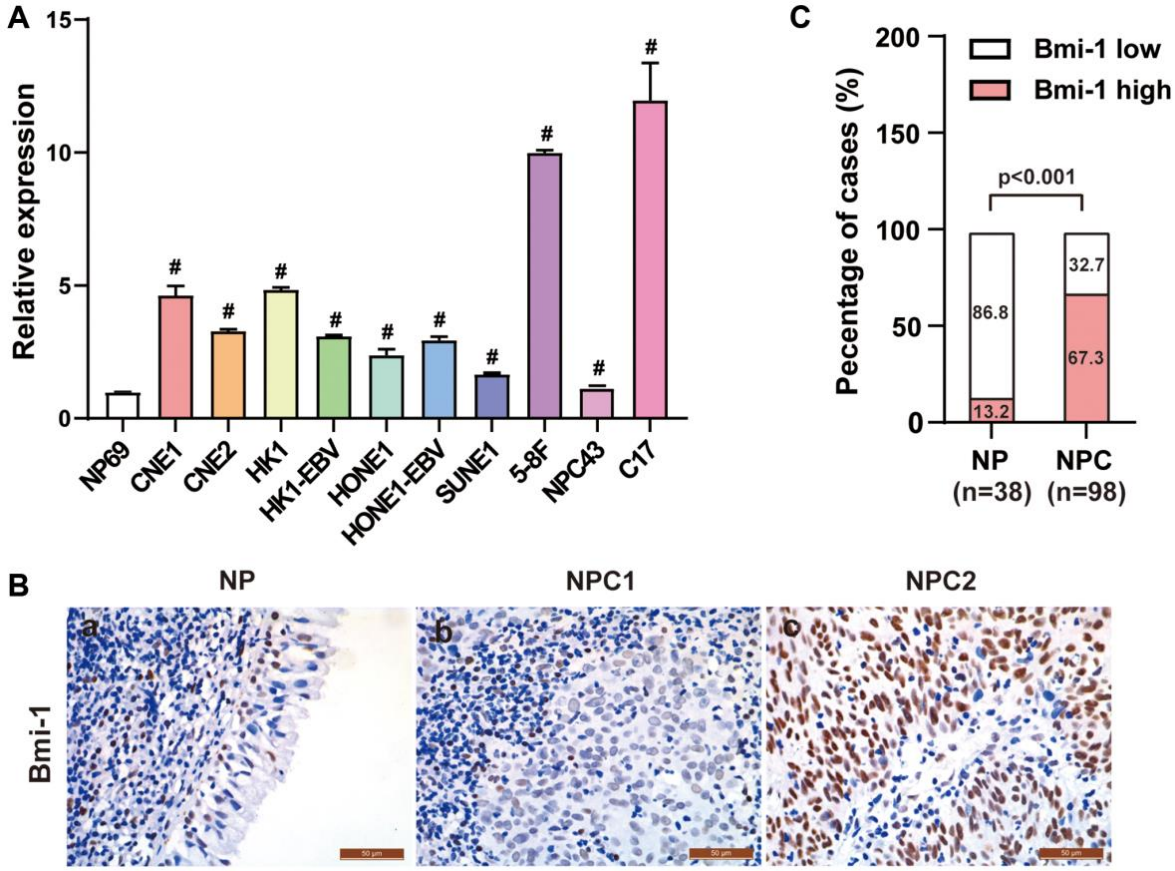
**Bmi-1 was markedly upregulated in NPC clinical tissue specimens.** (**A**) *Bmi-1* levels were detected using qRT-PCR in the NPC cell lines shown. (**B**) Representative photographs from immunohistochemical analyses of Bmi-1 protein levels in NPC tissues and non-cancerous nasopharyngeal epithelial tissues. (**C**) Bmi-1 levels were significantly greater in NPC tissues than in non-cancerous nasopharyngeal epithelial tissues (*P* < 0.001, χ^2^ test).

To assess the involvement of Bmi-1 in NPC progression, we used quantitative real-time PCR (qRT-PCR) to measure *Bmi-1* mRNA levels in NPC cell lines. *Bmi-1* was markedly upregulated in all the NPC cell lines we tested (CNE1, CNE2, HK1, HK1-EBV, SUNE1, HONE1, HONE1-EBV, 5-8F, NPC43 and C17) relative to NP69 nasopharyngeal epithelial cells (Figure 1A).

We then used immunohistochemical staining to examine Bmi-1 protein levels in archived paraffin-embedded tissue samples from 98 NPC biopsies and 38 non-cancerous nasopharyngeal biopsies. Low Bmi-1 levels were observed in 33 of the 38 non-cancerous nasopharyngeal epithelial samples (86.8%) (Figure 1B, 1C; Table 1). On the other hand, among the 98 NPC specimens, 32 (32.7%) exhibited low or non-detectable Bmi-1 expression, while 66 (67.3%) exhibited high Bmi-1 staining (Figure 1B, 1C; Table 1). Thus, high Bmi-1 expression was more common in NPC tissues than in non-cancerous nasopharyngeal tissues.

**Table 1:** Bmi-1 levels in 38 non-cancerous epithelial tissues and 98 NPC tissues.

**Tissue type**	***n***	**Bmi-1 expression**		**χ^2^**	***P***
		**High (*n*, %)**	**Low (*n*, %)**		
Non-cancerous epithelial tissues	38	5 (13.2)	33 (86.8)	32.226	<0.001
NPC	98	66 (67.3)	32 (32.7)		

### Bmi-1 upregulation is associated with aggressive NPC phenotypes

**Figure 2:**
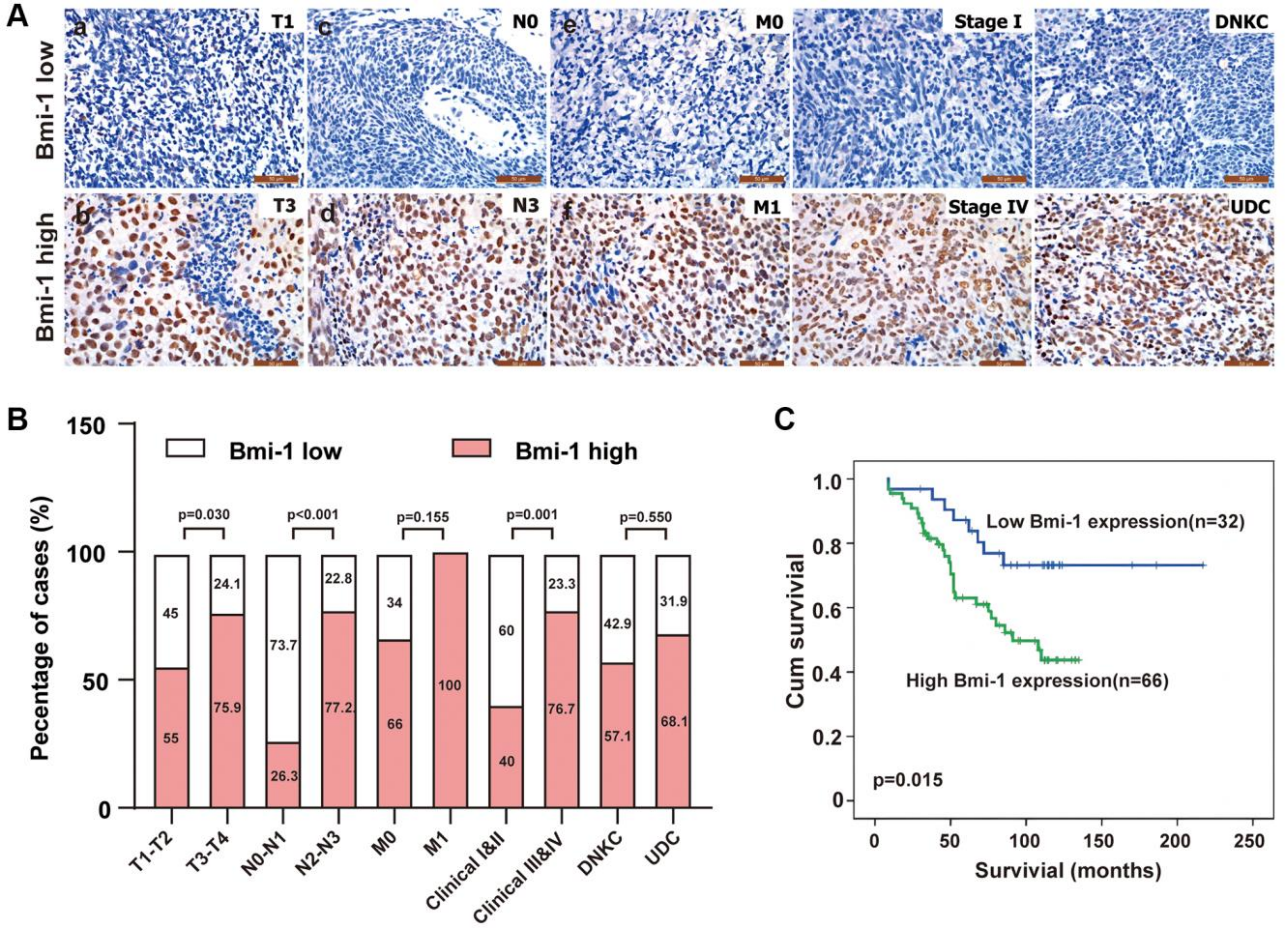
**Bmi-1 upregulation was associated with malignant tumor progression in NPC patients.** (**A**) Representative images of Bmi-1 levels in clinical tissue biopsies from NPC patients with differing tumor-node-metastasis (TNM) stages, clinical stages and histological types. Low Bmi-1 expression was detected in T1 (**a**), N0 (**c**), M0 (**e**), stage I (**g**) and differentiated nonkeratinizing carcinoma (DNKC) (**i**) NPC biopsies, while high Bmi-1 expression was observed in T3 (**b**), N3 (**d**), M1 (**f**), stage IV (**h**) and undifferentiated carcinoma (UDC) (**j**) tumors. (**B**) The number and percentage of samples with high and low Bmi-1 levels according to various clinicopathological traits (χ^2^ test). (**C**) Cumulative overall survival curves of 98 NPC patients with high or low Bmi-1 levels. A log-rank test was used to calculate the *P* value.

We then assessed the association of Bmi-1 levels with various clinicopathological traits in the 98 NPC patients (Table 2). Bmi-1 levels did not correlate with the age (*P* = 0.319) or sex (*P* = 0.221) of the NPC patients (Table 2). On the other hand, we did find positive correlations between Bmi-1 levels and the tumor size (‘T’; *P* = 0.030), lymph node invasion (‘N’; *P* < 0.001) and clinical stage (III-IV vs. I-II; *P* = 0.001) of NPC (Figure 2A, 2B; Table 2). Briefly, high Bmi-1 expression was detected more often in T3-T4, N2-N3 and stage III-IV NPC biopsies than in T1-T2, N0-N1 and stage I-II samples, respectively (Figure 2A, 2B; Table 2), demonstrating that the gain of Bmi-1 expression is an important feature of advanced NPC. In addition, overall survival was significantly shorter in patients with high Bmi-1 levels than in patients with low Bmi-1 levels (Figure 2C).

**Table 2:** Correlation of clinicopathological characteristics with Bmi-1 levels in NPC tissues.

**Characteristics**	**Case no.**	**Bmi-1 expression**		**χ^2^**	***P***
		**High (*n*, %)**	**Low (*n*, %)**		
Sex					
Female	26	15 (57.7)	11 (42.3)	1.500	0.221
Male	72	51 (70.8)	21 (29.2)		
Age (years)					
<47	53	38 (71.7)	15 (28.3)	0.994	0.319
≥47	45	28 (62.2)	17 (37.8)		
Histological type					
DNKC	7	4 (57.1)	3 (42.9)	0.357	0.550
UDC	91	62 (68.1)	29 (31.9)		
T classification					
T1-T2	40	22 (55)	18 (45)	4.685	0.030
T3-T4	58	44 (75.9)	14 (24.1)		
Lymph node metastasis					
N0-N1	19	5 (26.3)	14 (73.7)	18.044	<0.001
N2-N3	79	61 (77.2)	18 (22.8)		
Distant metastasis					
No	94	62 (66)	32 (34)	2.022	0.155
Yes	4	4 (100)	0 (0)		
Clinical stage					
I-II	25	10 (40)	15 (60)	11.414	0.001
III-IV	73	56 (76.7)	17 (23.3)		

Abbreviations: DNKC: differentiated nonkeratinizing carcinoma; UDC: undifferentiated carcinoma; T: tumor size; N: lymph node metastasis.

### Bmi-1 silencing suppresses NPC cell proliferation

**Figure 3:**
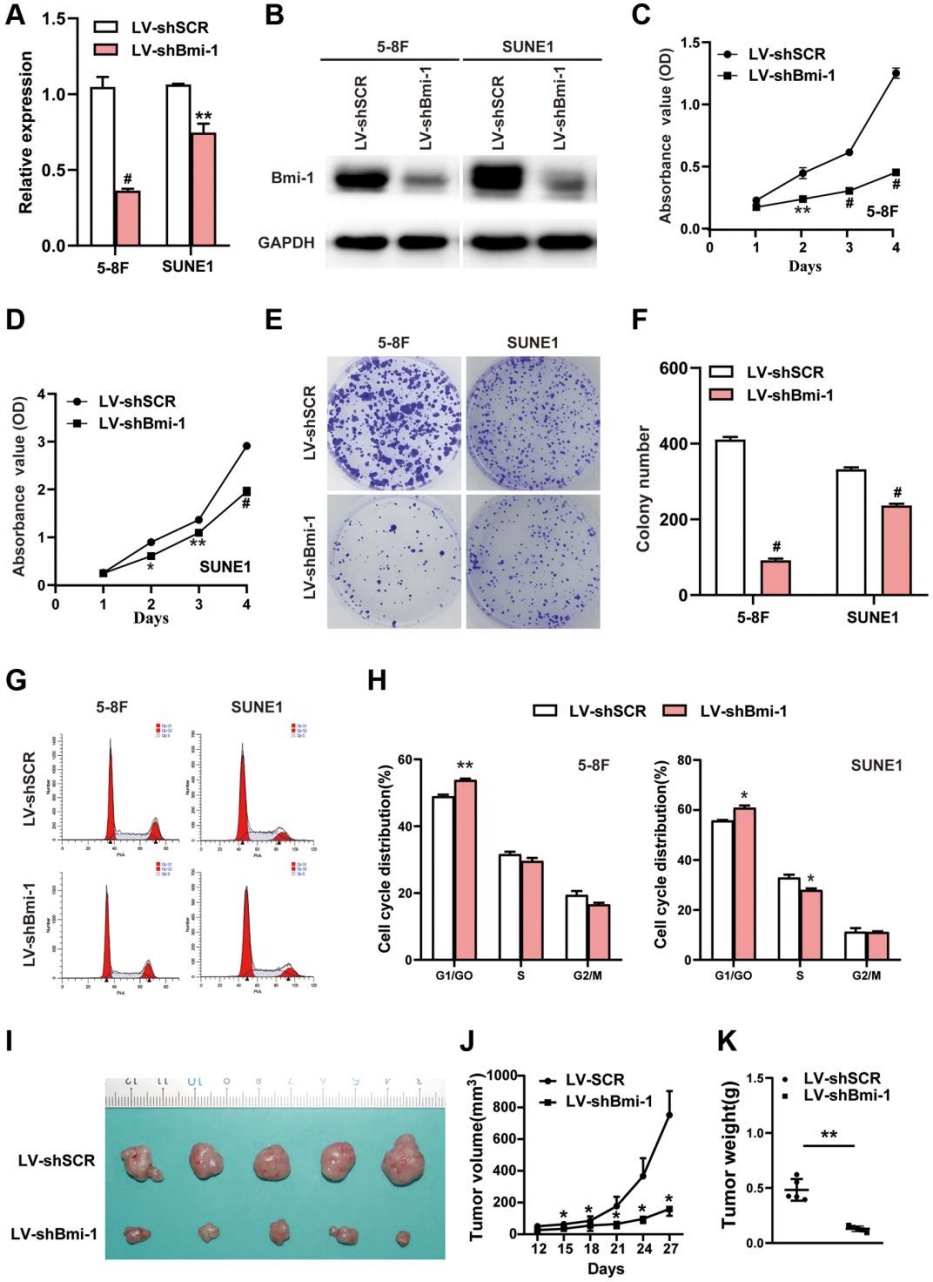
**RNAi-induced knockdown of Bmi-1 inhibited the *in vitro* proliferation and *in vivo* tumorigenesis of NPC cells.** (**A**) The relative mRNA levels of *Bmi-1* in shBmi-1-expressing 5-8F and SUNE1 cells were determined via qRT-PCR. SCR: scrambled control shRNA. (**B**) The protein levels of Bmi-1 in shBmi-1-expressing 5-8F and SUNE1 cells were determined via Western blotting. (**C**, **D**) A CCK8 assay was employed to assess the growth of shBmi-1-expressing 5-8F and SUNE1 cells. (**E**, **F**) A colony formation assay was used to examine the proliferation abilities of shBmi-1-expressing 5-8F and SUNE1 cells. (**G**, **H**) Propidium iodide staining and flow cytometry were used to detect the cell cycle distributions of shBmi-1-expressing 5-8F and SUNE1 cells (**G**), and the statistical results were calculated (**H**). (**I**–**K**) Bmi-1 knockdown inhibited tumor growth from 5-8F cells in nude mice. A representative tumor picture is shown (**I**), along with a tumor volume growth curve (**J**) and the tumor weights (**K**).

Given that Bmi-1 expression was significantly induced in NPC samples, we suspected that inhibiting Bmi-1 expression might suppress NPC progression. Thus, we used RNA interference (RNAi) with short hairpin RNA (shRNA) to evaluate whether knocking down Bmi-1 would diminish NPC cell growth. Compared with the scrambled control vector (shSCR), the Bmi-1 shRNA (shBmi-1) successfully repressed Bmi-1 mRNA (Figure 3A) and protein (Figure 3B) expression in 5-8F and SUNE1 cells. A Cell Counting Kit 8 (CCK8) assay revealed that silencing endogenous Bmi-1 inhibited the growth of 5-8F and SUNE1 cells (Figure 3C, 3D). Moreover, a colony formation assay indicated that shBmi-1 treatment notably reduced the number and size of colonies formed by 5-8F and SUNE1 cells (Figure 3E, 3F). Cell cycle analyses demonstrated that Bmi-1 knockdown induced cell cycle arrest at phase G1 (Figure 3G, 3H). Thus, the loss of Bmi-1 suppressed the proliferation of NPC cells *in vitro*.

### Bmi-1 knockdown reduces the tumorigenicity of NPC cells *in vivo*

We then examined the impact of Bmi-1 silencing on NPC cell growth *in vivo* by performing tumor xenograft experiments. Nude mice received subcutaneous injections of 5-8F cells expressing shSCR or shBmi-1. Tumors from mice injected with shSCR-expressing cells were notably larger in size (Figure 3I), volume (Figure 3J) and weight (Figure 3K) than those from mice injected with shBmi-1-expressing cells. These results demonstrated that Bmi-1 silencing suppressed the tumorigenicity of NPC cells *in vivo*.

### Knocking down Bmi-1 significantly reduces NPC cell stemness

**Figure 4:**
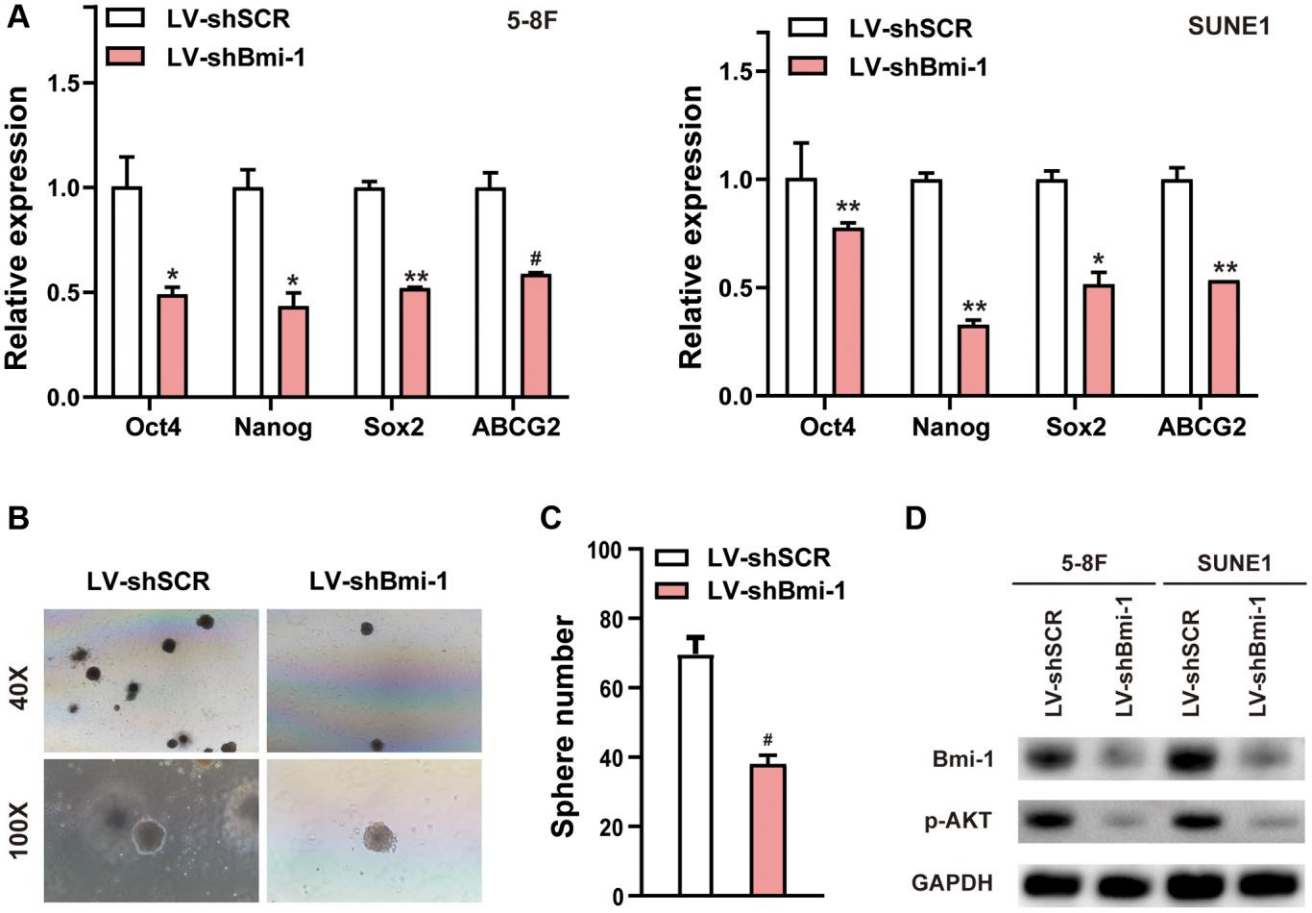
**RNAi-induced suppression of Bmi-1 reduced NPC cell stemness.** (**A**) qRT-PCR analysis of various genes in shBmi-1-expressing 5-8F and SUNE1 cells. (**B**, **C**) Depletion of endogenous Bmi-1 in 5-8F cells inhibited tumor sphere formation. (**D**) Western blotting results of cell extracts from shBmi-1-expressing 5-8F and SUNE1 cells. The loading control was GAPDH.

We next assessed stemness marker expression and tumor sphere formation to determine the effects of Bmi-1 inhibition on NPC stem cell-like populations. RNAi- induced depletion of endogenous Bmi-1 downregulated the mRNA levels of stem cell markers such as octamer-binding transcription factor 4 (*Oct4*), Nanog homeobox (*Nanog*), SRY-box transcription factor 2 (*Sox2*) and ATP binding cassette subfamily G member 2 (*ABCG2*) in NPC cells (Figure 4A). Sphere-forming assays revealed that silencing Bmi-1 diminished the number of spheres formed by NPC cells (Figure 4B, 4C). Furthermore, Western blotting indicated that pAKT levels were significantly reduced in shBmi-1-expressing NPC cells compared with shSCR-expressing NPC cells (Figure 4D). These findings illustrated that knocking down Bmi-1 reduced NPC cell stemness.

### Bmi-1 silencing inhibits the EMT, migration and invasion of NPC cells

**Figure 5:**
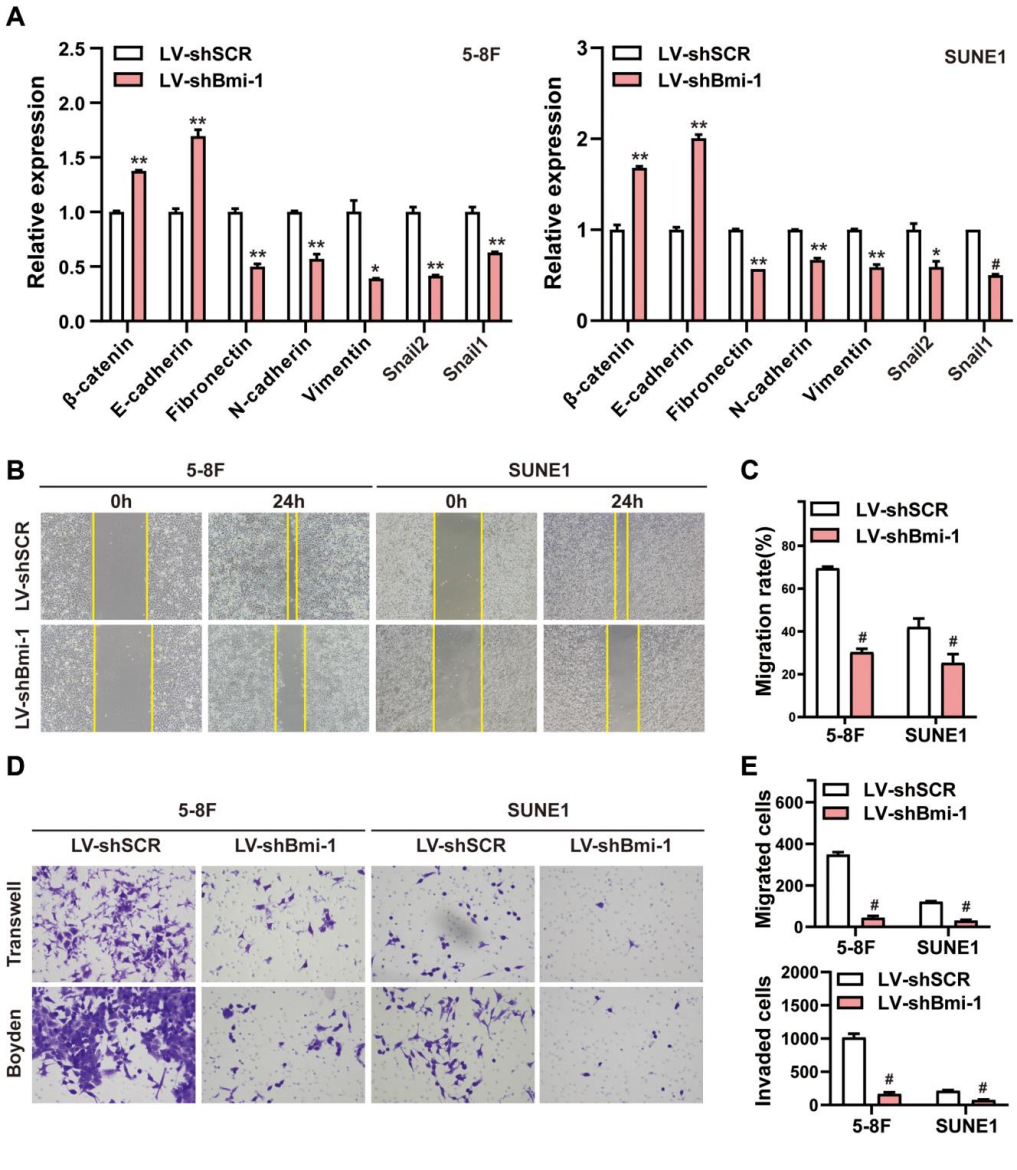
**RNAi-induced knockdown of Bmi-1 suppressed the EMT, migration and invasion of NPC cells *in vitro*.** (**A**) The mRNA levels of various genes in shBmi-1-expressing NPC cells were determined using qRT-PCR. (**B**, **C**) Wound healing assays were performed in shBmi-1-expressing 5-8F and SUNE1 cells. Migration activity was measured based on the distance from the scratch boundary lines to the cell-free space for 24 hours. (**D**, **E**) The motility and invasiveness of shBmi-1-expressing NPC cells were determined using Transwell migration and Boyden invasion assays, respectively.

Our results above demonstrated that Bmi-1 upregulation correlated with lymph node invasion and metastasis in human NPC samples (Figure 2 and Table 2). Considering that the EMT facilitates the invasion and metastasis of a variety of cancer types [45], we evaluated epithelial and mesenchymal marker expression after silencing endogenous Bmi-1 in NPC cells. Bmi-1 knockdown markedly upregulated epithelial markers (*E-cadherin* and *β-catenin*) and downregulated mesenchymal markers (*vimentin*, *N-cadherin*, *fibronectin*, *snail1* and *snail2*) at the mRNA level in 5-8F and SUNE1 cells (Figure 5A).

Then, we evaluated the impact of Bmi-1 on NPC cell mobility and invasiveness. Wound healing assays demonstrated that Bmi-1 silencing inhibited both 5-8F and SUNE1 cell migration (Figure 5B, 5C). Moreover, Transwell migration assays and Boyden chamber invasion assays indicated that Bmi-1 knockdown suppressed 5-8F and SUNE1 cell migration and invasion (Figure 5D, 5E). These results suggested that Bmi-1 silencing suppressed NPC cell motility and invasiveness by triggering events characteristic of the EMT *in vitro*.

### HRY binds to the promoter of *Bmi-1* to upregulate *Bmi-1* expression

**Figure 6:**
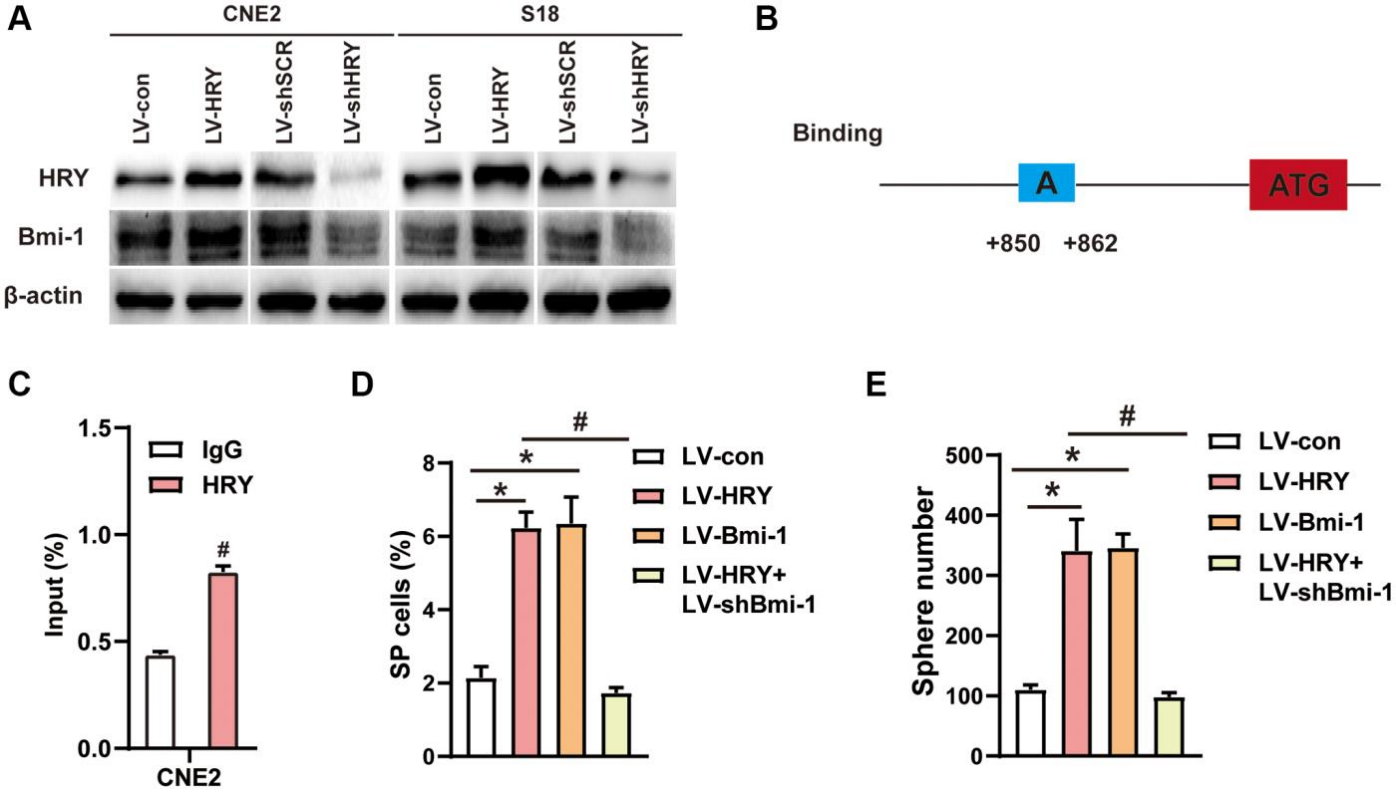
**HRY increased the stemness of NPC cells by promoting Bmi-1 expression.** (**A**) Western blotting was used to determine the protein levels of HRY and Bmi-1 in NPC cells transfected with different plasmids. (**B**) Schematic diagram of the *Bmi-1* promoter, displaying possible HRY binding sites. ATG: start codon for translation. (**C**) ChIP assays were conducted with anti-HRY or IgG antibodies to determine HRY binding sites on the *Bmi-1* promoter in CNE2 cells. (**D**) The proportions of SP cells among CNE2 cells transduced with different plasmids were analyzed using flow cytometry. (**E**) Tumor sphere formation in CNE2 cells transduced with different plasmids.

Our previous study [46] and other studies [47–50] have indicated that HRY/Hes1 enhances cancer cell stemness. Bioinformatic analyses predicted *Bmi-1* as a target of HRY. Therefore, we examined Bmi-1 expression in HRY-expressing or shHRY-expressing NPC cells. Western blotting revealed that HRY overexpression increased Bmi-1 levels, while HRY silencing significantly downregulated Bmi-1 (Figure 6A), suggesting that HRY enhances the expression of Bmi-1 in NPC cells. Then, we conducted a chromatin immunoprecipitation (ChIP) assay to assess the binding between HRY and *Bmi-1* in NPC cells. We found that HRY could bind to the *Bmi-1* promoter region at specific regulatory sequences (Figure 6B), and quantitative ChIP assays confirmed these findings (Figure 6C). These data suggested that HRY binds to the promoter of *Bmi-1* to induce the transcription of this gene.

### HRY enhances the stemness of NPC cells by upregulating Bmi-1

To understand whether the effects of Bmi-1 on NPC cell stemness depended on the upstream activity of HRY, we assessed the impact of gain and loss of HRY function. We performed side population (SP) cell detection and tumor sphere formation assays to evaluate the effects of exogenous HRY expression on stem cell-like populations in NPC. HRY overexpression (gain of function) notably increased the proportion of SP cells among CNE2 cells (6.2%, vs. 2.0% in the control; Figure 6D). Similar to the effects of exogenous Bmi-1 expression, HRY overexpression significantly increased the number of spheres formed by CNE2 cells (Figure 6E).

Subsequently, we investigated whether shBmi-1 could reverse the increase in stemness induced by ectopic HRY expression in NPC cells. We observed that shBmi-1 treatment prevented HRY overexpression from elevating the proportion of SP cells (Figure 6D) and increasing the sphere number and diameter (Figure 6E). These findings suggested that HRY induces NPC cell stemness by upregulating Bmi-1.

### Association between HRY and Bmi-1 levels in NPC patients

**Figure 7:**
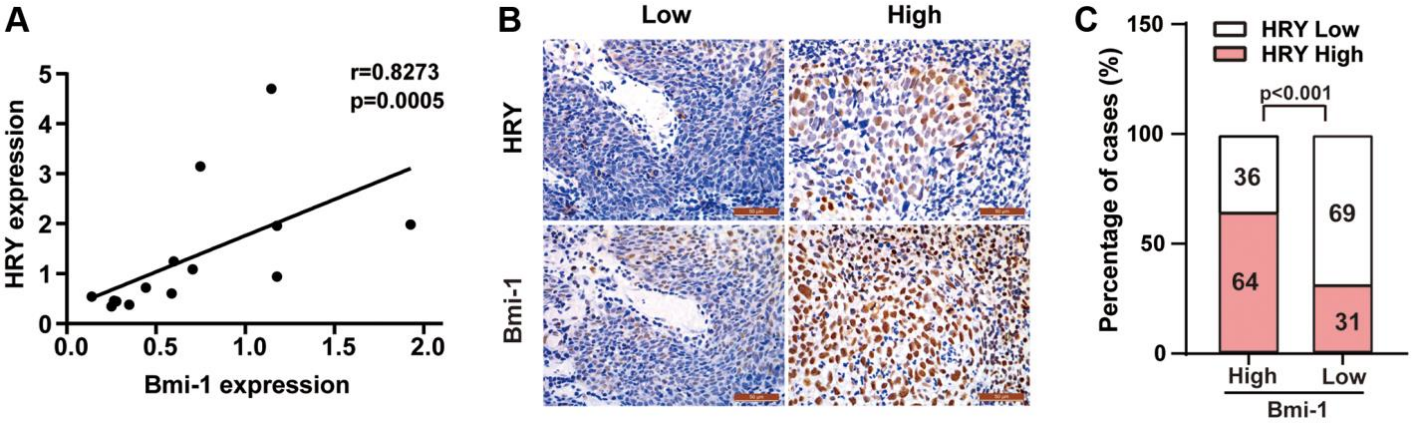
**HRY levels correlated positively with Bmi-1 levels in NPC tissues.** (**A**) *HRY* and *Bmi-1* mRNA levels correlated significantly and positively with one another in NPC samples (Spearman’s correlation analysis, r = 0.8273, *P* = 0.0005). (**B**) Relationship between HRY and Bmi-1 levels in immunohistochemical analysis of NPC tissues. (**C**) HRY levels correlated positively with Bmi-1 levels in NPC tissues (χ^2^ test).

Our earlier results indicated that Bmi-1 was upregulated in tissues from NPC patients (Figure 1B, 1C; Table 1). Moreover, we previously reported that HRY expression was elevated in NPC specimens [51]. Thus, we assessed the correlation between Bmi-1 and HRY levels in NPC specimens using qRT-PCR and immunohistochemistry. We observed a significant positive association between *Bmi-1* and *HRY* mRNA levels in NPC biopsies (two-tailed Spearman’s correlation, r = 0.8273, *P* = 0.0005; Figure 7A). Likewise, we detected a correlation between Bmi-1 and HRY protein levels in NPC tissues (Figure 7B, 7C). Thus, HRY levels correlated positively with Bmi-1 levels at both the mRNA and protein levels in NPC specimens.

## DISCUSSION

NPC, a malignant cancer of the head and neck, is distinctly distributed among ethnic groups and localities worldwide [1–3]. More NPC patients die from distant metastases than from their primary tumors [1–3]. However, it is unclear which molecular pathways cause NPC to progress in malignancy.

Bmi-1 is considered to be oncogenic, as it contributes to the progression of various cancers and is upregulated in glioma, colorectal cancer, breast cancer and prostate cancer [4–6]. Here, we observed that Bmi-1 levels were significantly elevated in NPC cell lines and tissue specimens compared with their normal counterparts. More importantly, higher Bmi-1 expression tended to be detected in clinical stage III-IV, T3-T4 and N2-N3 patient samples than in stage I-II, T1-T2 and N0-N1 samples, respectively. We subsequently demonstrated that silencing Bmi-1 suppressed NPC cell proliferation, stemness, motility and invasion, which was consistent with these findings from the previously published studies [26, 52]. Thus, evidence from NPC cells, tissues and functional experiments clearly illustrated that Bmi-1 is an oncogene contributing to the development of NPC.

Bmi-1 maintains and/or promotes the self-renewal of cancer stem-like cells/tumor-initiating cells in a variety of tumors [9–21], and thus is an ideal target for treatments aimed at cancer stem-like cells. For example, Bmi-1 inhibition in colorectal cancer [12] and combined suppression of Bmi-1 and enhancer of zeste 2 Polycomb repressive complex 2 subunit (EZH2) expression in glioma [15] effectively eliminated cancer stem-like cells, thereby achieving an ideal anticancer efficacy. Various highly selective small molecules (e.g., PTC-028, PTC-209 and PTC596) have been developed to inhibit Bmi-1 for basic and clinical tumor treatment, and the results have been encouraging [12, 53–59]. In this study, we observed that Bmi-1 silencing remarkably suppressed the stemness of NPC cells.

We then demonstrated that *Bmi-1* may be transcriptionally induced by HRY. Dramatic upregulation of HRY has been noted in various cancer types, including breast cancer [60], colon cancer [46, 61, 62], glioma [63], head and neck squamous cell carcinoma [64], hepatocellular carcinoma [65], lung cancer [66], medulloblastoma [67], meningioma [68], NPC [51] and ovarian carcinoma [69]. Our previous study [46] and other studies [46–50, 70] have also indicated that HRY enhances cancer cell proliferation and stemness, further illustrating the oncogenic activity of HRY. The present study revealed that Bmi-1 may promote the proliferation and stemness of NPC cells as a downstream target of HRY. HRY levels correlated positively with Bmi-1 levels in NPC tissues, and HRY induced Bmi-1 expression in NPC cells. Moreover, HRY was bound to the *Bmi-1* promoter, suggesting that *Bmi-1* is a bona fide transcriptional target of HRY.

Subsequently, we investigated whether HRY increased the stemness of NPC cells *in vitro* by inducing Bmi-1. We observed that HRY overexpression increased NPC cell stemness in a manner similar to ectopic Bmi-1 expression, whereas shBmi-1 treatment reversed the increased stemness in HRY-expressing CNE2 cells. These results demonstrated that HRY reinforces the stemness of NPC cells by upregulating its target gene, *Bmi-1*.

Despite these findings, the mechanism(s) by which Bmi-1 silencing suppresses NPC progression remain unclear. A previous study revealed that Bmi-1 transcriptionally repressed the tumor suppressor phosphatase and tensin homolog (*PTEN*), thereby activating the phosphoinositide 3-kinase (PI3K)/AKT pathway, inducing the EMT and promoting the invasion and metastasis of NPC cells [23]. The suppression of PTEN has been shown to activate the PI3K/AKT/glycogen synthase kinase 3β pathway in various kinds of cancer cells, thus greatly promoting their proliferation and stemness [71–77]. These data suggest that Bmi-1 may enhance the proliferation and stemness of NPC cells by inducing PTEN/PI3K/AKT signaling, although further investigation is needed to confirm this.

In conclusion, our study revealed that Bmi-1 downregulation suppressed tumor progression during the pathogenesis of NPC. Thus, Bmi-1 could be a useful treatment target in advanced NPC patients. However, studies combining ChIP-seq and RNA-seq assays are needed to identify the downstream target genes responsible for the tumor-promoting effects of this transcription factor, and these results should be verified in subsequent validation and functional studies.

## MATERIALS AND METHODS

### Cell lines and cell culture

The human NPC (5-8F, C17, CNE1, CNE2, HK1, HK1-EBV, HONE1, HONE1-EBV, NPC43 and SUNE1) and immortalized nasopharyngeal epithelial (NP69) cells were kind gifts from Prof. Qiao Tao (Chinese University of Hong Kong, Hong Kong, China), Prof. GSW Tsao (University of Hong Kong), Prof. Yixin Zeng (Sun Yat-sen University, Guangzhou, China) and Prof. Musheng Zeng (Sun Yat-sen University). The cells were maintained at 37°C in a humidified incubator containing 5% CO_2_. RPMI 1640 medium supplemented with 10% fetal bovine serum was used to culture the NPC cells, while keratinocyte/serum-free medium (Invitrogen) was used for the NP69 cells.

### Clinical specimens

Tissue punches were generated from formalin-fixed, paraffin-embedded tumor specimens obtained from patients diagnosed with primary NPC at the Department of Pathology, Sun Yat-Sen University Cancer Center and at the Department of Pathology, the Second Affiliated Hospital of Guilin Medical University in Guilin, China. The tissue microarray was produced using non-cancerous nasopharyngeal epithelial tissue punches and NPC tissue punches. Histopathological information was collected from pathology reports, and survival data were collected in raw form from the patients’ attending physicians. Tissue and clinical data were retrieved according to the regulations of the Sun Yat-Sen University Cancer Center institutional review board and of the Second Affiliated Hospital of Guilin Medical University institutional review board, and data safety laws concerning ethical standards and patient confidentiality. The application of the tissue microarray was approved by the Medical Ethics Committee of Sun Yat-Sen University Cancer Center and the Second Affiliated Hospital of Guilin Medical University.

### Immunohistochemistry

Tissue microarray blocks were cut into 4-μm sections, and then were deparaffinized and rehydrated. Antigen retrieval was performed by treating the sections with high pressure for 3 minutes in sodium citrate buffer. Subsequently, 3% H_2_O_2_ was used to block the sections for 10 minutes, and an overnight incubation was performed with a primary antibody against Bmi-1 (Proteintech, cat. no. 66161-1-Ig) at 4°C. A goat anti-rabbit secondary antibody was then used to stain the slides for 20 minutes at 37°C. Lastly, diaminobenzidine staining and hematoxylin counterstaining were performed. The intensity of the staining was graded as 0 (negative), 1 (weak), 2 (moderate) or 3 (strong), with scores of 0 or 1 being defined as low, and scores of 2 or 3 being considered high. Two pathologists who were blinded to the patients’ clinicopathological data independently evaluated the results of the histopathological and immunohistochemical studies.

### RNA isolation and qRT-PCR

RNA isolation, reverse transcription and qRT-PCR were conducted as reported previously [78–85]. The qRT-PCR primers are detailed in Supplementary Table 1. Glyceraldehyde 3-phosphate dehydrogenase (*GAPDH*) was employed as an endogenous control. Internal controls were used to normalize all samples, and relative quantification (2^−ΔΔCt^) was used to calculate fold-changes.

### Plasmids, lentivirus production and lentiviral transduction for stable cell lines

The oligonucleotide used to knock down human *Bmi-1* was GTTCACAAGACCAGACCAC (‘shBmi-1’). The lentiviral shRNA construct for human *Bmi-1* was obtained according to the pLenti-U6-GFP-Puro vector protocol. For lentivirus production, 293T cells were co-transfected with lentiviral packaging plasmids (psPAX2 and pMD2G; Addgene) and the lentiviral vectors, as reported previously [78–85]. Then, 5-8F and SUNE1 cells were infected with the lentiviruses. The pWPXL-HRY plasmid was supplied by Addgene (Addgene plasmid 36983). Prof. Ryoichiro Kageyama (Kyoto University, Kyoto, Japan) kindly provided the pCSII vectors containing the scrambled and HRY-knockdown sequences.

### Western blotting

Proteins were lysed, electrophoretically separated on sodium dodecyl sulfate polyacrylamide gels, and transferred to polyvinylidene difluoride membranes. Primary antibodies against Bmi-1 (Proteintech, cat. no. 66161-1-Ig; mouse, 1:1000 dilution), HRY (Abcam, cat. no. ab71559; mouse, 1:2000 dilution), GAPDH (Proteintech, cat. no. 10494-1-AP; rabbit, 1:5000 dilution) or β-actin (Proteintech, cat. no. 81115-1-RR; rabbit, 1:5000 dilution) were used to probe the membranes. Then, the membranes were incubated with horseradish peroxidase-labeled secondary antibodies. Enhanced chemiluminescence was used to detect the hybridization signals. The loading control was either GAPDH or β-actin. Supplementary Table 2 lists the antibodies used in the present study.

### CCK8 and colony formation assays

The CCK8 assay (cat. no. CK04, Dojindo, Japan) and colony formation assay were conducted as reported previously [78–85].

### Cell cycle analysis

The cell cycle analysis was conducted as reported previously [78, 81].

### Tumor xenografted mice

Three- to four-week-old male BALB/c nude mice were obtained from the Experimental Animal Center of Southern Medical University and provided with autoclaved drinking water and laboratory rodent chow. The left dorsal thigh of each mouse (*n* = 6) was injected subcutaneously with 2.5 × 10^6^ shSCR- or shBmi-1-expressing 5-8F cells. Daily monitoring was performed, and a caliper slide rule was used to measure the tumor volume. The tumor volume was determined as 1/2 (width^2^ × length). The mice were euthanized on the 27th day after transplantation. These experiments were performed with strict adherence to the Guide for the Care and Use of Laboratory Animals of Southern Medical University. The Committee on the Ethics of Animal Experiments of Southern Medical University approved the animal protocol.

### Wound healing assay

The wound healing assay was conducted as reported previously [81].

### Transwell migration and Boyden invasion assays

The Transwell migration and Boyden invasion assays were conducted as reported previously [51, 79, 81, 82, 85–87].

### ChIP

In CNE2 cells, HRY binding sites on the *Bmi-1* promoter were identified using a ChIP assay in accordance with the manufacturer’s instructions. In brief, formaldehyde (1% final concentration) was used for cross-linking during a 10-minute incubation at room temperature, and a glycine solution was used to terminate the reaction. Then ice-cold phosphate-buffered saline containing 0.1 mM phenylmethylsulfonyl fluoride was used to wash the cells. The cells were centrifuged at 1000 rpm for 5 minutes, and 1 mL of ChIP sonication buffer was used to resuspend the cell pellet. Sonication was performed to shear the DNA, and a 3-minute centrifugation at 9,000 × *g* was used to pellet the cell debris. Equal aliquots of the chromatin supernatants were immunoprecipitated with an anti-HRY (Abcam) or IgG (negative control) antibody overnight. The ChIP-qPCR primers are shown in Supplementary Table 3.

### Flow cytometry analysis of the percentages of SP cells

Trypsin (0.25%) was used to digest the NPC cells, and then two washes with calcium/magnesium-free phosphate-buffered saline were performed. Ice-cold RPMI 1640 medium supplemented with 2% fetal bovine serum was used to resuspend the cells to a concentration of 1 × 10^6^ cells/mL. The cells were placed an incubator containing 5% CO_2_ at 37°C for 90 minutes. Then, flow cytometry was used to evaluate the percentage of SP cells.

### Statistical analysis

All data are shown as the mean ± standard deviation. SPSS 16.0 software was used for statistical analyses. The association of clinicopathological traits with Bmi-1 expression was assessed using a χ^2^ test. A log-rank test was used to analyze the cumulative overall survival. Spearman’s correlation analysis was performed to determine the correlation between *Bmi-1* and *HRY* levels. A two-tailed Student’s *t*-test was used to compare two independent groups (^*^P < 0.05, ^**^P < 0.01, ^#^*P* < 0.001).

## Supplementary Materials

Supplementary Tables
