# Supplementary material for: Hairy gene homolog increases nasopharyngeal carcinoma cell stemness by upregulating Bmi-1
Source: Aging (Albany NY). 2023 May 22;15(10):4391–410. doi: 10.18632/aging.204742 (PMC10258013; doi:10.18632/aging.204742)
Supplement: Supplementary Tables [file aging-15-204742-s001.pdf]

SUPPLEMENTARY TABLES

Supplementary Table 1. Primers for qRT-PCR analysis.

Gene	Forward primer (5′–3′)	Reverse primer (5′–3′)
GAPDH	ACCCAGAAGACTGTGGATGG	TCTAGACGGCAGGTCAGGTC
Oct4	CTTGCTGCAGAAGTGGGTGGAGGAA	CTGCAGTGTGGGTTTCGGGCA
Sox2	GCCGAGTGGAAACTTTTGTCG	GGCAGCGTGTACTTATCCTTCT
ABCG2	AGCAGCTCTTCGGCTTGCAACA	GTTCCAACCTTGGAGTCTGCCACT
β-catenin	AGGTCTGAGGAGCAGCTTCA	ATTGTCCACGCTGGATTTTC
E-cadherin	TGCCCAGAAAATGAAAAAGG	GTGTATGTGGCAATGCGTTC
Fibronectin	CAGTGGGAGACCTCGAGAAG	TCCCTCGGAACATCAGAAAC
N-cadherin	ACAGTGGCCACCTACAAAGG	CCGAGATGGGGTTGATAATG
Vimentin	GAGAACTTTGCCGTTGAAGC	GCTTCCTGTAGGTGGCAATC
Snail 1	CACTATGCCGCGCTCTTTC	GCTGGAAGGTAAACTCTGGATTAGA
Snail 2	ACTCCGAAGCCAAATGACAA	CTCTCTCTGTGGGTGTGTGT

Supplemental Table 2. List of antibodies and suppliers used in the study.

Antibody	Isotype	Suppliers	Cat. no	Application
GAPDH	Rabbit IgG	Proteintech	10494-1-AP	WB
Bmi-1	Mouse/IgG2b	Proteintech	66161-1-Ig	WB, IHC
HRY	Mouse	abcam	ab71559	WB, IHC
β-actin	Rabbit	Proteintech	81115-1-RR	WB
p-AKT	Rabbit	Cell Signaling Technology	4060L	WB, IHC, IP

Supplementary Table 3. Primers used in ChIP assays.

Gene	Forward primer (5′–3′)	Reverse primer (5′–3′)
Bmi-1	AGGCGGCATGAGACGAGC	GGGCGGAAAAGACAATGAAAG
